# Supplementary material for: Pyridinone Derivatives as Interesting Formyl Peptide Receptor (FPR) Agonists for the Treatment of Rheumatoid Arthritis
Source: Molecules. 2021 Oct 30;26(21):6583. doi: 10.3390/molecules26216583 (PMC8587000; doi:10.3390/molecules26216583)
Supplement: Supplementary file 1 [file molecules-26-06583-s001.zip › molecules-1433642-supplementary.pdf]

Supplementary Materials for

Pyridinone Derivatives as Interesting Formyl Peptide Receptor (FPR) Agonists for the Treatment of Rheumatoid Arthritis

Letizia Crocetti [1], Claudia Vergelli [1], Gabriella Guerrini [1], Maria Paola Giovannoni [1,*], Liliya N. Kirpotina [2], Andrei I. Khlebnikov [3,4], Carla Ghelardini [5], Lorenzo Di Cesare Mannelli [5], Elena Lucarini [5], Igor A. Schepetkin [2] and Mark T. Quinn [2]

[1]NEUROFARBA, Pharmaceutical and Nutraceutical Section, University of Florence, Via Ugo Schiff 6, 50019 Sesto Fiorentino, Italy.
[2]Department of Microbiology and Cell Biology, Montana State University, Bozeman, MT, USA
[3]National Research Tomsk Polytechnic University, 634050 Tomsk, Russia
[4]Scientific Research Institute of Biological Medicine, Altai State University, 656049 Barnaul, Russia
[5]NEUROFARBA, Pharmacology and Toxicology Section, University of Florence, Viale Pieraccini 6, 50139 Florence, Italy.
* Correspondence: mariapaola.giovannoni@unifi.it; Tel +39-055-4573682; Fax +39-055-4573780

A)

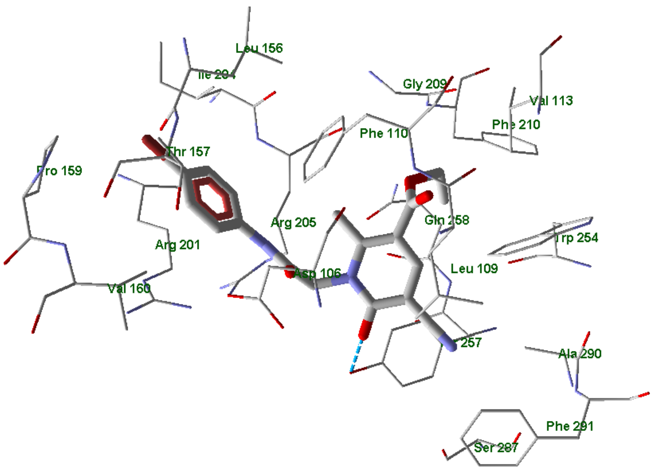

B)

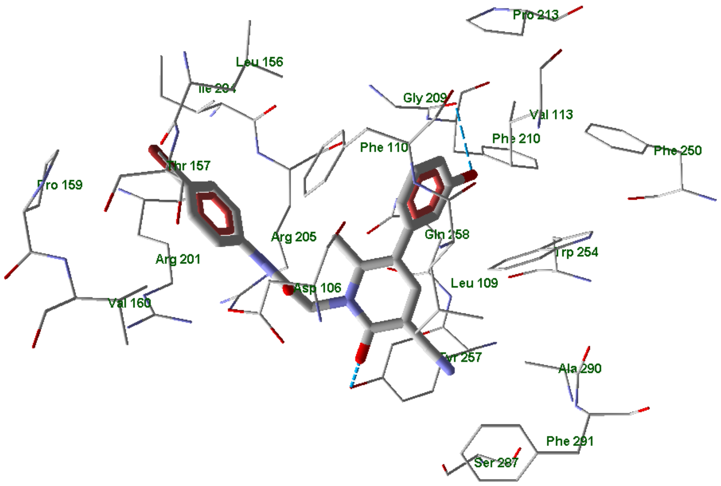

C)

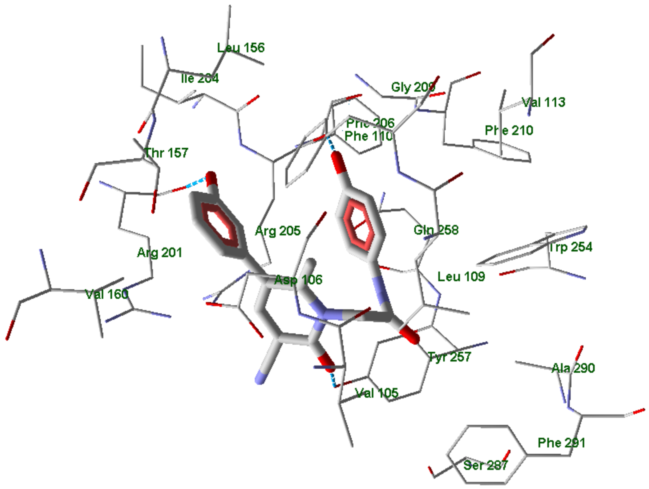

Figure S1. Docking poses of compounds **AMC4** (Panel A), **14a** (Panel B), and **14c** (Panel C) in FPR1 binding site. Residues within 4 Å from each pose are visible. Hydrogen bonds are shown in dashed blue lines. All the compounds are H-bonded to Tyr257 residue. Additionally, compound **14a** forms a hydrogen bond with Gly209, while **14c** is H-bonded to Arg201 and Arg205.

A)

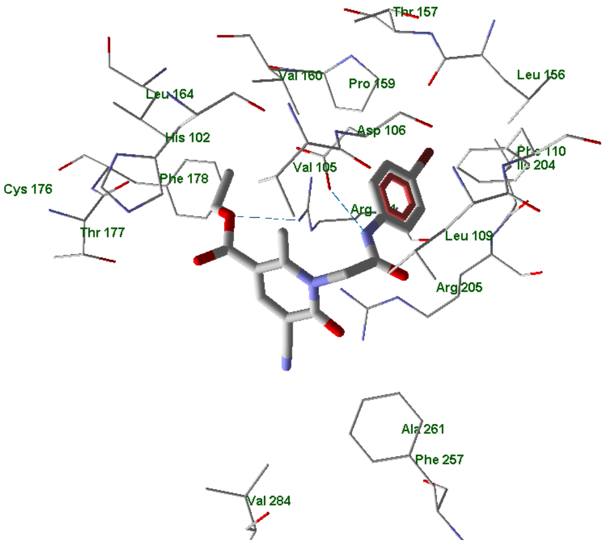

Thr 157
Leu 156
Val 160
Pro 159
Leu 164
His 102
Asp 106
Phe 110
Ile 204
Cys 176
Phe 178
Val 105
Thr 177
Leu 109
Arg 205
Ala 261
Phe 257
Val 284

B)

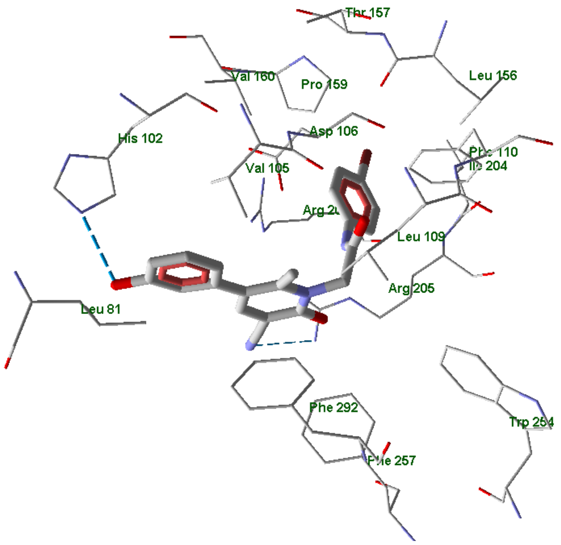

Thr 157
Val 160
Pro 159
Leu 156
His 102
Asp 106
Phe 110
Ile 204
Val 105
Leu 109
Arg 205
Leu 81
Phe 292
Trp 264
Phe 257

C)

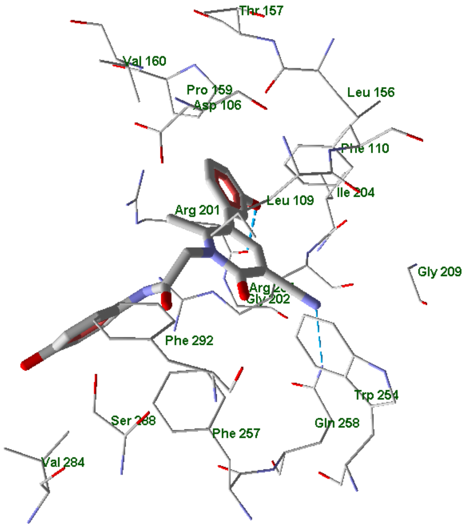

Figure S2. Docking poses of compounds **AMC4** (Panel **A**), **14a** (Panel **B**), and **14c** (Panel **C**) in FPR2 binding site (PDB entry 6OMM). Residues within 4 Å from each pose are visible. Compound **14a** forms hydrogen bonds with His102 and Arg205. Compounds **AMC4** and **14c** are H-bonded to Arg201 residue. Additionally, **AMC4** and **14c** form H-bonds with Asp106 and Gln258, respectively. Hydrogen bonds are shown in dashed blue lines.
